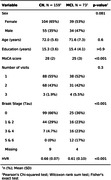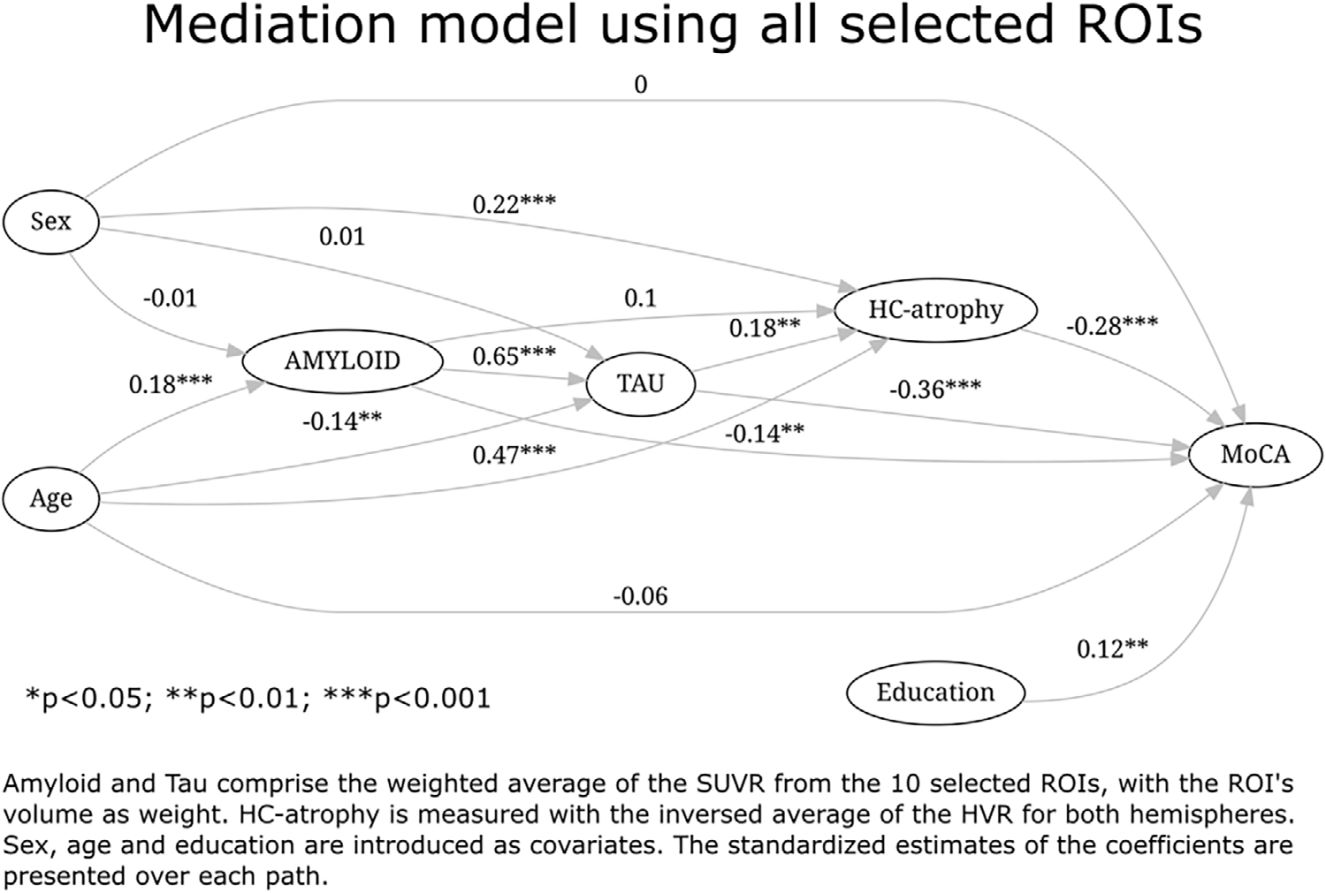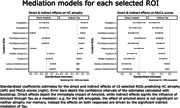# Tau accumulation mediates β‐amyloid effects on hippocampal atrophy and memory in early Alzheimer’s disease

**DOI:** 10.1002/alz.094070

**Published:** 2025-01-09

**Authors:** Sofia Fernandez‐Lozano, Vladimir S Fonov, Joseph Therriault, Nesrine Rahmouni, Stijn Servaes, Jenna Stevenson, Nina Marguerita Poltronetti, Pedro Rosa‐Neto, D Louis Collins

**Affiliations:** ^1^ McConnell Brain Imaging Centre, Montreal Neurological Institute, McGill University, Montreal, QC Canada; ^2^ Translational Neuroimaging Laboratory, The McGill University Research Centre for Studies in Aging, Montréal, QC Canada; ^3^ Translational Neuroimaging Laboratory, The McGill University Research Centre for Studies in Aging, Montreal, QC Canada; ^4^ Department of Biomedical Engineering, McGill University, Montreal, QC Canada

## Abstract

**Background:**

While we know dementia in Alzheimer’s disease (AD) results from the accumulation of ß‐amyloid (Aß), tau pathology, and hippocampus atrophy (HC), it is still unclear how these factors impose cognitive decline.

**Method:**

We used Structural Equation Models (SEM; lavaan R package) to explore the complex relationships between the neurobiological factors in the early stages preceding AD dementia in the TRIAD cohort. Our sample comprised 333 timepoints of neuropsychological evaluation (MoCA), structural MRI, Aß ([18F]AZD4694), and tau ([18F]MK6240) PET of cognitively healthy (NC) and mild cognitive impaired (MCI) participants. We measured neurodegeneration with an inverted HC‐to‐Ventricle ratio (HVR), i.e. 1‐HVR, an integrity measure composed of the ratio of the HC volume and the sum of the volumes of HC and the temporal horn of the lateral ventricle. We used a feature selection algorithm (Boruta R package) to select the most important Regions of Interest (ROIs) from the CerebrA atlas relating Aß & Tau to memory. We then used SEM to explore the mediation of Aß and Tau on HC‐atrophy and memory using 1) the average Aß & Tau SUVR of all ROIs and 2) separate models using the Aß & Tau SUVR for each of the ROIs. Age, sex and education were treated as covariates. For ease of comparison, we calculated the standardized coefficients in our models.

**Result:**

The demographic data of our sample is presented in Table 1. The selected ROIs were: bilateral amygdala, hippocampus, and parahippocampal gyrus; right fusiform gyrus and orbitofrontal cortex (lateral and medial portions); and left middle temporal gyrus. The full SEM with all ROIs show full mediation between Aß, Tau and HC‐atrophy, and partial mediation between all biomarkers (Fig. 1). On the distinct models by ROI, Amyloid’s effect on MoCA is almost always indirect through Tau (Fig. 2).

**Conclusion:**

We show that, even in the early stages of AD, Aß aggregation alone has no effect on HC atrophy, and only a weak effect on cognitive decline; interactions with tau pathology is required to modulate the effects of Aß aggregation on both HC atrophy and memory loss.